# Porous Carbon Spheres (PCSs) as a Support for Ni Nanoparticles for Alkaline Water Electrolyzer Cathodes

**DOI:** 10.3390/molecules31183308

**Published:** 2026-09-18

**Authors:** Ahmed A. A. Almaazmi, Galal A. Nasser, Lantao Liu, Jan Kopyscinski, Sasha Omanovic

**Affiliations:** Department of Chemical Engineering, McGill University, 3610 University Street, Montreal, QC H3A 0C5, Canada; galal.nasser@mail.mcgill.ca (G.A.N.); liulantaogood@163.com (L.L.); jan.kopyscinski@mcgill.ca (J.K.)

**Keywords:** alkaline water electrolysis, hydrogen production, nickel nanoparticles, porous carbon spheres

## Abstract

Porous carbon spheres (PCSs) were synthesized as a support for nickel nanoparticles (Ni NPs) to fabricate a cathode for hydrogen evolution reaction (HER) in an alkaline electrolyte. Characterization of the material revealed that both non-activated and activated PCS exhibited a porous, spherical morphology without pronounced structural confinement, while the deposited Ni NPs showed a tendency to aggregate into large clusters with an average particle size ranging from 16 nm to 22 nm, depending on the type of the PCS used (non-activated vs. activated). X-ray diffraction and Raman spectroscopy confirmed the predominantly amorphous and defect-rich nature of both pristine (PCS) and activated carbon support (PCS-ACT), providing favorable anchoring sites for metal nanoparticles. Although pristine PCS displayed a higher specific surface area than PCS-ACT (284 ± 10 vs. 143 ± 5 m^2^ g^−1^, respectively), the surface area of the surface-immobilized Ni NPs was comparable, yielding 5.0 ± 0.3 m^2^ of Ni per gram of the composite. Despite the comparable surface area of the immobilized Ni nanoparticles, electrochemical measurements demonstrated that Ni–PCS outperformed Ni–PCS-ACT in terms of HER electrocatalytic activity. This behavior was attributed to improved accessibility of active Ni sites and reduced pore blockage by evolved hydrogen due to the larger pore diameter of the Ni–PCS material. Thus, these findings suggest that optimizing the pore structure and mass-transport characteristics may be as important as enhancing metal dispersion when designing porous carbon-supported Ni electrocatalysts for alkaline water electrolysis.

## 1. Introduction

Global energy consumption has increased in the past decade, with a shift in energy supply from coal to oil and natural gas. As a result of the use of primary energy sources, carbon dioxide concentrations have increased dramatically. In the 1960s, CO_2_ annual emissions from burning fossil fuels were 11 billion tons compared to the current emissions of 18.3 to 37.0 billion tons of CO_2_ into the atmosphere. Also, building oil refineries and drilling require large amounts of land, which impacts ecosystems and landscapes. Furthermore, coal, oil, and natural gas pollute waterways and groundwater and contribute to ocean acidification, which threatens marine life. Given the current world energy concerns of using fossil fuels, new approaches to clean energy solutions are being used to reduce CO_2_ emissions [1,2].

Solar, wind, geothermal, and hydroelectric energy are all forms of renewable energy that have been installed and used in various locations. Renewable resources produce large amounts of surplus energy that can be stored in batteries. Green hydrogen gas produced by water electrolysis using a surplus of renewable energy is becoming a popular option for storing substantial amounts of energy, as well as for the green production of various products (ammonia, steel, to name a few). However, to make hydrogen production commercially viable, new and efficient electrode materials are needed (among other important requirements).

There are three major water electrolysis technologies: alkaline, proton/polymer-exchange membrane (PEM), and anion-exchange membrane (AEM) electrolyzers, with the PEM and AEM offering significant advantages over the alkaline water electrolysis (AWE), especially for the use with intermittent renewable energy sources [1,3,4,5,6].

AEM water electrolysis (AEMWE) is a cost-effective alternative to PEM electrolysis, as it allows the use of non-precious transition metal catalysts such as nickel, which is not the case with PEM electrolyzers that use platinum and iridium as an electrocatalyst. The goal of AEMWE is to combine the low cost of alkaline electrolysis with the high power and flexibility of PEM electrolysis [7,8].

In both AEM and PEM electrolyzers, carbon (mostly carbon black) is used as a cathode electrocatalyst support [9,10]. It not only minimizes the agglomeration of electrocatalyst nanoparticles and improves electrical conductivity, but it also affects the electronic structure of the electrocatalyst nanoparticles through interfacial interactions. Various forms of carbon supports have been tested: Yang et al. [11] decorated nickel nanoparticles on carbon fiber cloth (Ni NPs/CFC) for HER in an alkaline medium. The authors concluded that the structure of the Ni NPs on CFC strengthened the mechanical adhesion and reduced interfacial resistance between the electrocatalysts and CFC substrates, enabling faster electron transport and effective electronic collection. Chen et al. [12] fabricated Ni on carbon nanotubes with a wool ball-like morphology (Ni-CNTs) for cathodic hydrogen evolution reaction in alkaline media. The findings have shown that the Ni-CNT cathode exhibited the strongest electrocatalytic activity among the samples produced. Ai et al. [13] encapsulated nickel nanoparticles on ultrathin graphene layers (Ni@graphene) as a promising candidate for hydrogen generation in 1M KOH. It was suggested that the remarkable HER efficiency of Ni@graphene was due to the catalytic sites of metallic Ni and the intact metal shielding effect of the outer graphene layers. A research route led by Mahale et al. [14] explored the HER performance of nickel nanoparticles distributed evenly on graphene sheets by electrophoretic deposition (EPD) in an alkaline solution, which demonstrated enhanced HER activity of the produced cathodes. Peng et al. [15] employed a hollow carbon sphere support and decorated it with ruthenium (Ru) structures encapsulated in the cavity, resulting in an excellent HER performance in alkaline media. Collectively, these studies demonstrate that carbon supports play an active role in determining the performance of Ni-based HER catalysts rather than serving solely as electrically conductive substrates. Depending on their structure and surface chemistry, carbon materials can modify nanoparticle dispersion, electronic properties, active-site accessibility, and mass transport. However, these effects are often coupled, making it difficult to establish a direct relationship between specific support properties and catalytic activity.

In this work, we synthesized and used porous carbon spheres (PCSs) with regular spherical, porous architectures as supports for nickel nanoparticles (Ni NPs) to produce cathode catalysts for the hydrogen evolution reaction (HER) in an alkaline electrolyte [16]. Structural characterization and electrochemical tests demonstrate that the catalytic performance of the cathode does not simply rely on the Ni specific surface area. Instead, the optimized pore structure of a PCS effectively improves the accessibility of electrolyte to Ni active sites by alleviating pore blockage induced by in situ generated hydrogen bubbles during electrolysis and optimizes the interfacial mass transport, thereby enhancing the overall electrocatalytic performance. These findings confirm that the rational regulation of pore architecture and gas–liquid mass-transport capability is equally crucial to improving metal nanoparticle dispersion and the electrolyte-accessible specific surface area in the design of high-performance carbon-supported non-precious metal electrocatalysts for alkaline water electrolysis, offering new insights into the structural optimization of advanced electrocatalysts for energy conversion.

## 2. Results and Discussion

### 2.1. Materials Characterization

**Figure 1:**
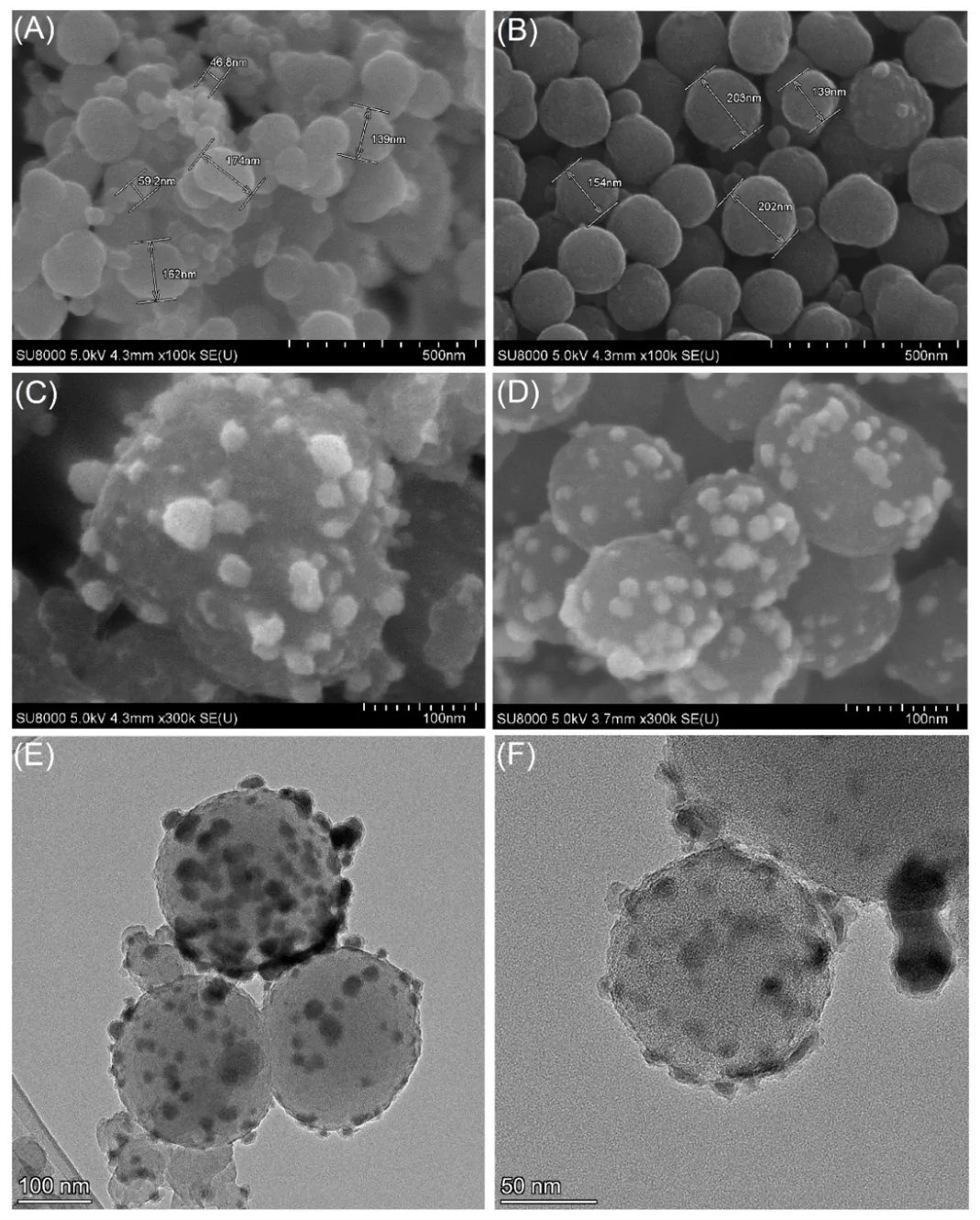
SEM micrographs of (**A**) PCS, (**B**) PCS-ACT, (**C**) Ni-PCS, (**D**) Ni-PCS-ACT, and TEM micrographs of (**E**) Ni-PCS and (**F**) Ni-PCS-ACT.

To explore the morphology of the synthesized PCS substrates and nickel catalyst immobilized on PCS, SEM images were recorded (Figure 1). The SEM image of PCSs in Figure 1A shows well-defined, roughly spherical particles. The surface of the spheres appears relatively smooth but with a slight granular texture typical of amorphous carbon or polymer-derived carbon structures. The particles are not isolated but are instead aggregated. There is visible “necking” or fusion between adjacent spheres, particularly between the smaller and larger particles. This interconnectivity is often advantageous for electrical conductivity in electrochemical applications, as it provides continuous pathways for electron transport, but disadvantageous in terms of the decreased specific surface area. There is a population of larger spheres with diameters ranging roughly from 140 nm to 175 nm. Interspersed among the larger spheres are much smaller satellites or secondary particles, with diameters of ca. 45–60 nm. This size variation can be beneficial for packing density. Namely, smaller particles can fill the interstitial voids between larger spheres, potentially increasing the specific (volumetric) surface area, which is beneficial for electrode materials.

The activation of PCS results in the change in the morphology of the PCS, as shown in Figure 1B for PCS-ACT. A particle size distribution ranges from ca. 140 nm to 200 nm. Notably, larger spheres (ca. 200+ nm) are present, whereas they were absent in the non-activated sample (Figure 1A). Unlike the PCS sample, the image of the PCS-ACT sample displays a more uniform size distribution: the population of smaller particles (ca. 45–60 nm) is drastically reduced (only a few could be seen), and the sample is dominated by the larger primary spheres. The surface of the PCS-ACT spheres appears slightly rougher and more irregular compared to the pristine PCS, and while the PCS-ACT particles remained generally spherical, they appear slightly more “faceted” or distorted than the smooth PCSs in Figure 1A, which is the result of the carbon surface activation.

Figure 1C shows Ni nanoparticles immobilized on PCSs. The Ni nanoparticles appear as bright, high-contrast spots, often forming irregular clusters and large aggregates on the smooth surface of the carbon spheres. The particle size distribution of Ni nanoparticles on PCSs displays a broad, skewed profile (Figure S1A), indicating a wide range of particle sizes, with an average diameter of 18 nm. This may suggest that during the growth of Ni nanoparticles on PCSs, agglomeration occurred due to insufficient defect sites or functional groups on the PCS surface to strongly anchor the metal precursor. Conversely, Ni nanoparticles on the PCS-ACT substrate show a slightly different size distribution (Figure S1B), characterized by a bimodal distribution at around 16 nm and 22 nm. This indicates that the PCS-ACT surface likely contains surface sites with varying surface energies.

The TEM images in Figure 1E,F offer evidence of the internal structure of the carbon spheres and the shape of the immobilized Ni nanoparticles. The images of the carbon spheres do not show a large, distinctly outlined central void. Instead, the entire volume of the spherical carbon exhibits a porous network (evidenced by BET measurements and discussed later in the manuscript), appearing as a heterogeneous, light gray texture. The Ni nanoparticles, seen as dark, high-contrast features, are highly localized and tend to cluster on the carbon spheres. Some Ni nanoparticles appear to be embedded within the spheres rather than on their surfaces. This embedding likely results from the natural porosity of the carbon spheres, indicating that the nickel precursor infiltrated the pores before reduction to form metallic nickel. Despite the high specific surface area of the carbon spheres (discussed later), the Ni nanoparticles tend to form large clusters, matching the broad size distribution also seen in the SEM and particle size distribution images (Figure 1C,D and Figure S1A,B, respectively). This clustering arises from the low density of stable anchoring points on the carbon surface, which promotes the migration and agglomeration of nickel atoms during the growth of Ni nanoparticles.

**Figure 2:**
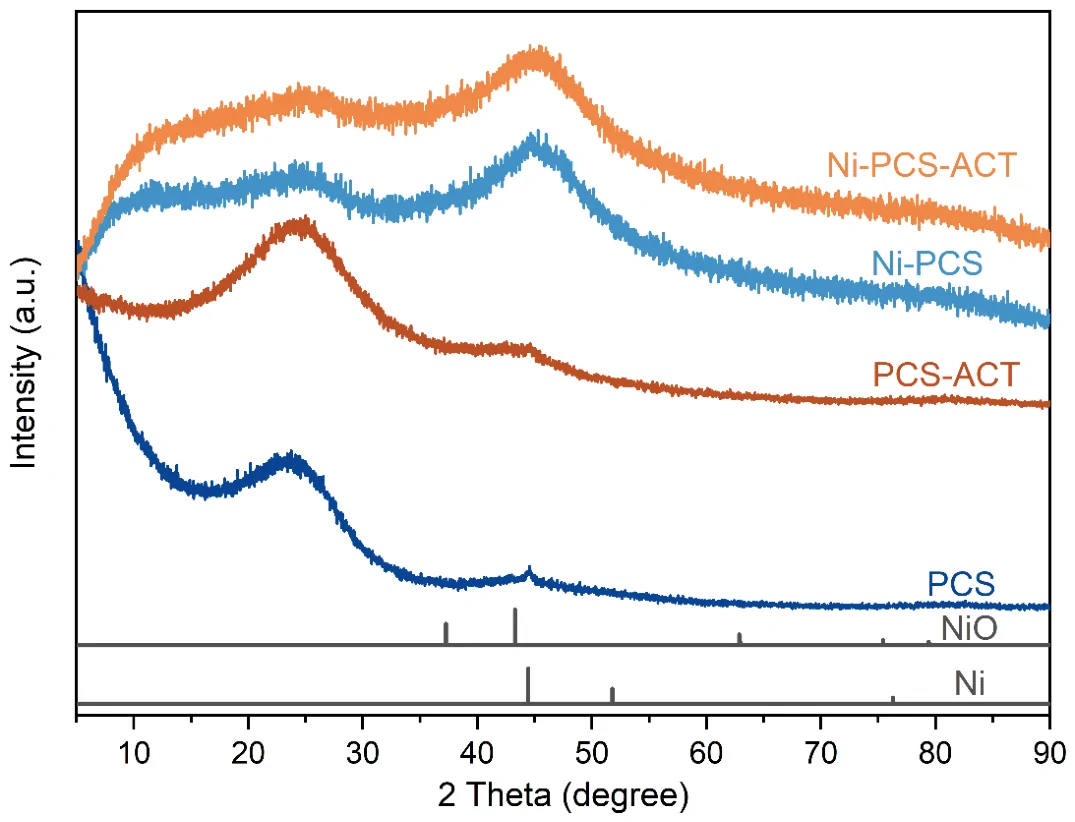
XRD patterns of PCS, activated PCS (PCS-ACT), Ni on PCS (Ni-PCS), and Ni on activated PCS (Ni-PCS-ACT). Reference patterns for Ni and NiO are PDF #04-010-6148 and PDF#00-044-1159, respectively.

To assess the crystalline structure of PCS and Ni nanoparticles, XRD spectra (Figure 2) were recorded for all the samples investigated. Firstly, both PCS and PCS-ACT show broad peaks at 25° and 43°, associated with 022 and 100 graphite, respectively [17]. These two broad, poorly defined peaks indicate the presence of disordered porous and amorphous structures in carbon spheres. Furthermore, Ni-PCS-ACT and Ni-PCS both exhibited a very broad peak at 44.5°, indicating an amorphous structure and is consistent with contributions from Ni and/or NiO phases [18,19,20]. An additional contribution to broadening could be attributed to the presence of Ni on PCS as nanoparticles [21,22]. The selected area electron diffraction (SEAD) results (Figure S2 in the Supporting Materials) also confirmed the amorphous structure of the Ni nanoparticles.

**Figure 3:**
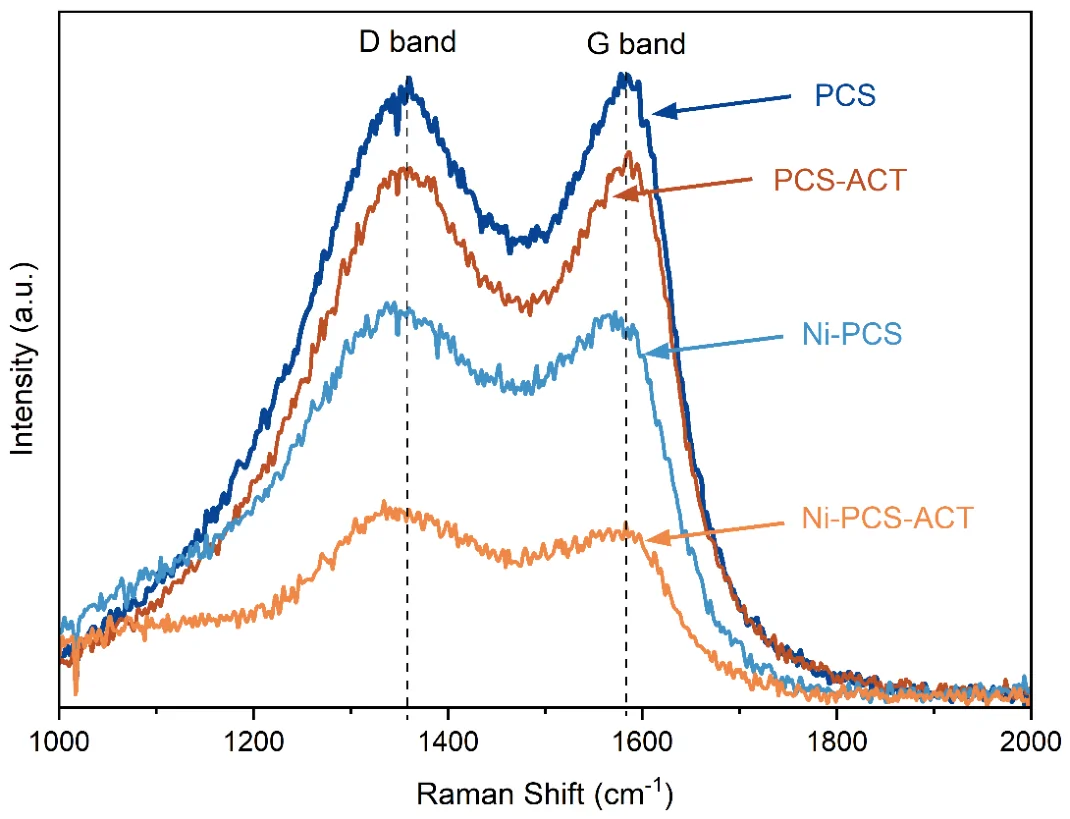
Raman spectra of PCS, activated PCS (PCS-ACT), Ni on PCS (Ni-PCS), and Ni on activated PCS (Ni-PCS-ACT).

The Raman spectrometry analysis of the samples studied in this work (Figure 3) reveals that PCS, PCS-ACT, Ni-PCS, and Ni-PCS-ACT are characterized by the presence of D (1350 cm^−1^) and G (1560 cm^−1^) bands, indicating the presence of defects or disorders [19,23]. Namely, the G band corresponds to the E_2g_ mode of graphite, reflecting the presence of graphitic carbon. In contrast, the D band can be related to disordered carbon structures, such as sp^3^ carbons, vacancies, dangling bonds, and foreign atoms embedded in the graphitic layers. This characteristic becomes more pronounced in the presence of structural and topological defects. The D band intensity depends on in-plane displacements, leading to a loss of hexagonal symmetry within the two-dimensional graphite lattice [24]. The ratio of the G and D band intensities indicates the degree of crystallinity [25], and the intensity ratios of the D and G bands (*I_D_*/*I_G_*) for Ni-PCS-ACT, Ni-PCS, PCS-ACT, and PCS are 1.105, 1.025, 0.971, and 0.997, respectively. Thus, XRD and Raman both indicate that carbon spheres are amorphous and exhibit a high degree of structural defects.

**Figure 4:**
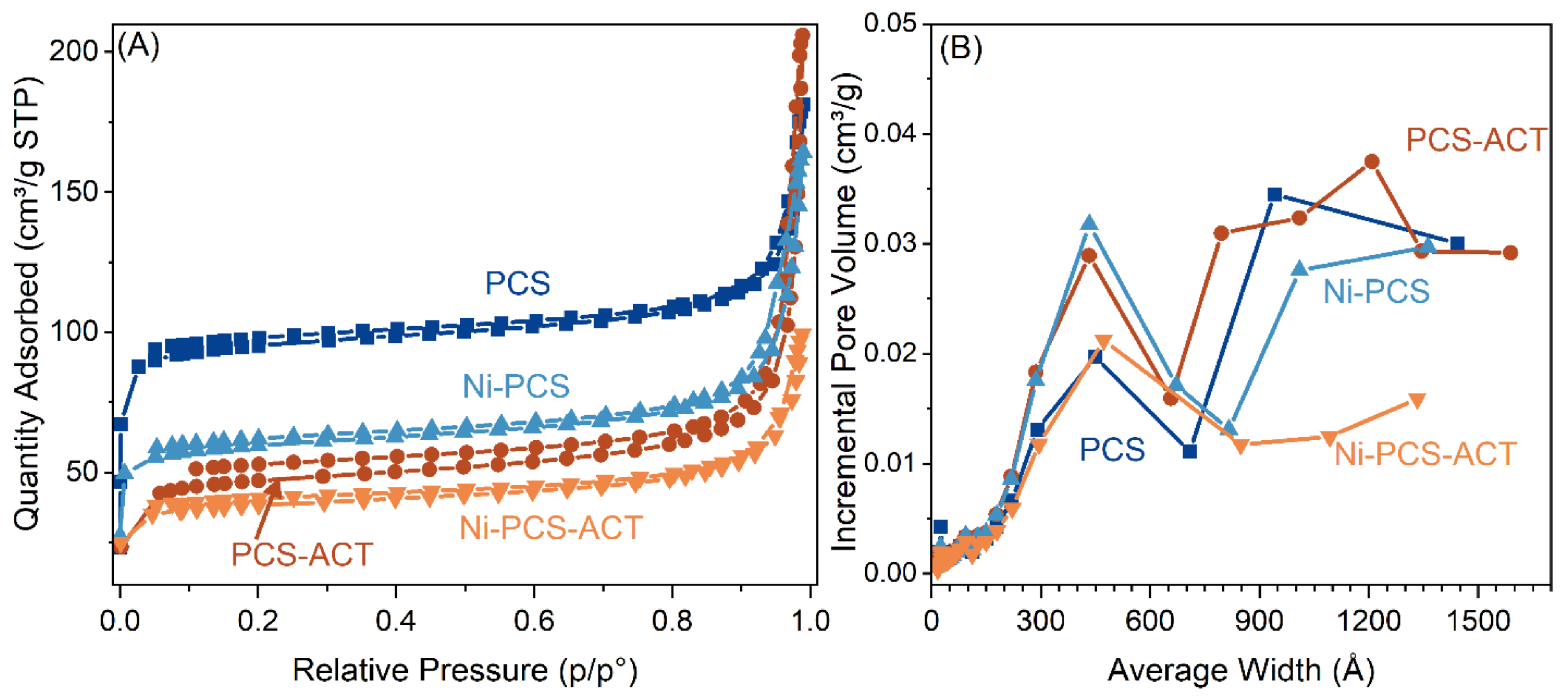
(**A**) Nitrogen adsorption/desorption isotherms and (**B**) pore-size distribution of PCS, activated PCS (PCS-ACT), nickel on PCS (Ni-PCS), and nickel on activated PCS (Ni-PCS-ACT).

To characterize the specific surface area and porosity of the synthesized samples, nitrogen adsorption–desorption experiments were performed, as shown in Figure 4. The corresponding textural parameters determined from the isotherms are summarized in Table 1. As evidenced in Figure 4A, all four samples exhibit composite Type I/IV adsorption–desorption behavior [26]. The nearly vertical increase in N_2_ uptake at low relative pressures (*p*/*p*^0^ < 0.05) indicates micropore filling and represents a Type I contribution, consistent with the literature [27,28]. This is followed by a gradual increase in adsorption at intermediate relative pressures, which is associated with monolayer–multilayer adsorption and resembles Type II behavior. In addition, the hysteresis and pronounced uptake at high relative pressures indicate capillary condensation in mesopores, characteristic of Type IV behavior, together with the filling of larger pores and interparticle voids near saturation. The absence of a limiting plateau at high relative pressures is consistent with an H3-like hysteresis loop, which may arise from irregular pores, a broad pore-size distribution, and interparticle voids [26]. This composite micro–meso–macroporous structure is consistent with the pore-size distributions presented in Figure 4B and the micropore and meso–macropore volumes reported in Table 1.

The specific surface area of PCS is 284 m^2^ g^−1^, which is lower than those reported for hollow hierarchical porous carbon spheres calcined at 1000 °C, denoted as HPCS-1000 in reference [29] (411 m^2^ g^−1^), carbon black Vulcan XC presented in reference [30] (460 m^2^ g^−1^), and mesoporous carbon spheres reported in reference [31] (1207 m^2^ g^−1^). After PCS activation, the surface area of PCS-ACT decreased to 143 m^2^ g^−1^, although its total pore volume (*V_T_*) and average pore diameter (*D_P_*) increased from 0.28 to 0.32 cm^3^ g^−1^ and from 4.0 to 8.9 nm, respectively. This change was accompanied by a decrease in micropore volume from 0.12 to 0.05 cm^3^ g^−1^ and an increase in the meso–macropore volume from 0.16 to 0.27 cm^3^ g^−1^, suggesting that activation reduced the contribution of micropores while promoting the widening or development of larger pores. Accordingly, 43% of the PCS total pore volume was occupied by micropores, while the remaining 57% corresponded to mesopores and macropores. For PCS-ACT, the micropore and meso–macropore contributions were 16% and 84%, respectively.

On the other hand, the surface areas and pore volumes of Ni-PCS and Ni-PCS-ACT decreased when Ni was immobilized on or within the carbon spheres, probably due to the partial occupation or blocking of the accessible pore surface by the metal species. To illustrate, the *S_BET_* values of Ni-PCS and Ni-PCS-ACT were 182 and 117 m^2^ g^−1^, representing reductions of 36% and 18% relative to PCS and PCS-ACT, respectively. However, the reduction in *V_T_* for Ni-PCS relative to PCS was much smaller (11%) than that observed for Ni-PCS-ACT relative to PCS-ACT (53%). This difference suggests that Ni immobilization affected the larger pores of PCS-ACT more strongly, consistent with the decrease in its average pore diameter from 8.9 to 5.3 nm. Furthermore, Ni-PCS had a larger *V_T_* and *D_P_* than Ni-PCS-ACT, which, as discussed later in the manuscript, is beneficial for the electrocatalytic activity of the material toward the hydrogen evolution reaction. Moreover, Ni-PCS and Ni-PCS-ACT exhibited almost identical micropore-to-total pore volume ratios *V_micro_*/*V_T_* of 28% and 27%, respectively.

The H_2_ pulse chemisorption measurements (Figure S3) were used to determine the nickel active surface area. Both Ni-PCS and Ni-PCS-ACT have similar H_2_ uptakes of 126.8 and 130.0 µmol g^−1^, respectively, and as such have the same active surface of around 5 m^2^ g^−1^ (Table 2). By comparing the specific surface area values in Table 1 and Table 2, it could be observed that the relative surface coverage of PCS and PCS-ACT by nickel is very small, yielding 2.7% of the PCS surface covered by Ni and 4.3% of the PCS-ACT surface covered by Ni. The larger relative surface coverage of PCS-ACT by Ni is probably due to the activation of PCS, thus enabling more Ni NP anchoring surface sites. Nevertheless, the specific active surface area of Ni is the same in both cases (Table 2).

**Figure 5:**
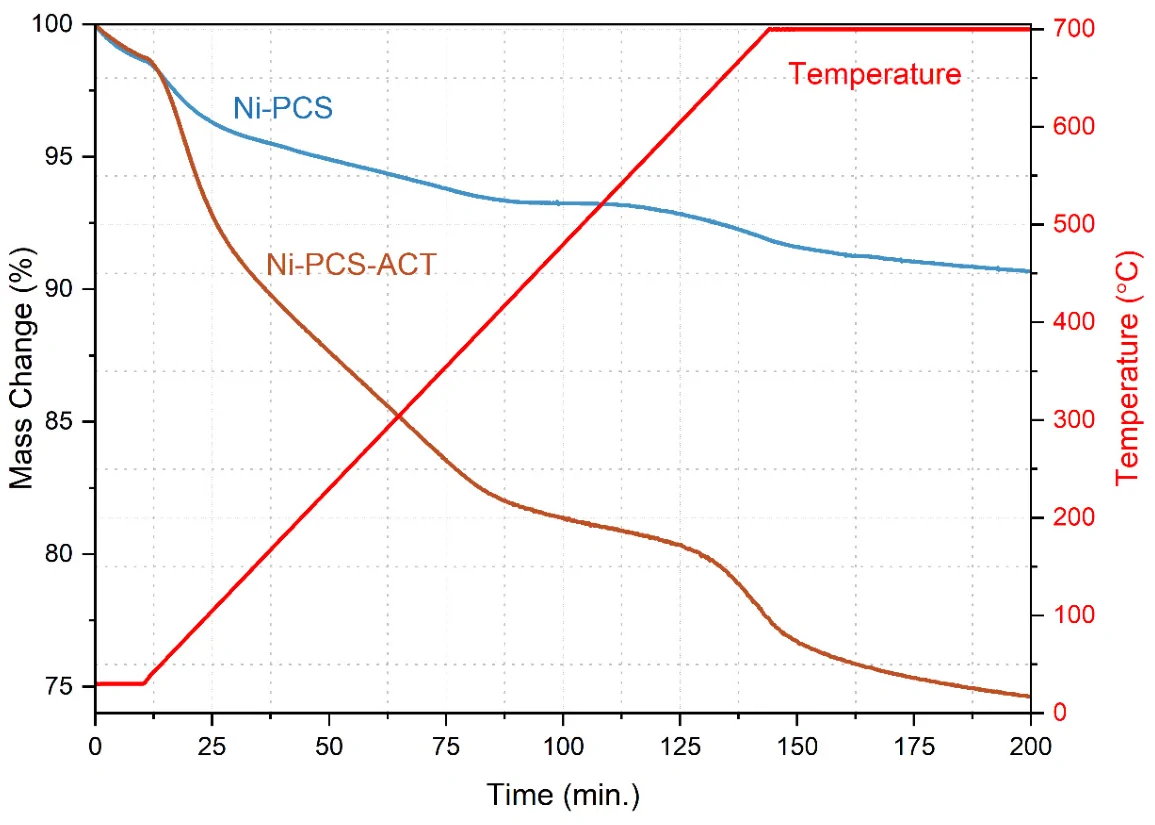
TGA profile of the Ni-PCS and Ni-PCS-AC.

TGA of the Ni-PCS and Ni-PCS-ACT materials was performed under an inert argon atmosphere to study their thermal stability. The results, presented in Figure 5, show sequential mass-loss regions that reflect mainly two segments of the materials’ decomposition. The initial mass loss up to ~400 °C is attributed to the removal of water and residual solvents. This kind of mass loss is common in Ni-loaded activated carbons, which occurs below 300 °C [32]. A further gradual mass decrease between 400 and 630 °C suggests decomposition of surface functional groups and volatile carbon. Notably, Ni-PCS-ACT displayed a larger and steeper mass loss compared to Ni-PCS, indicating a reduced thermal stability.

### 2.2. Electrochemical Characterization

**Figure 6:**
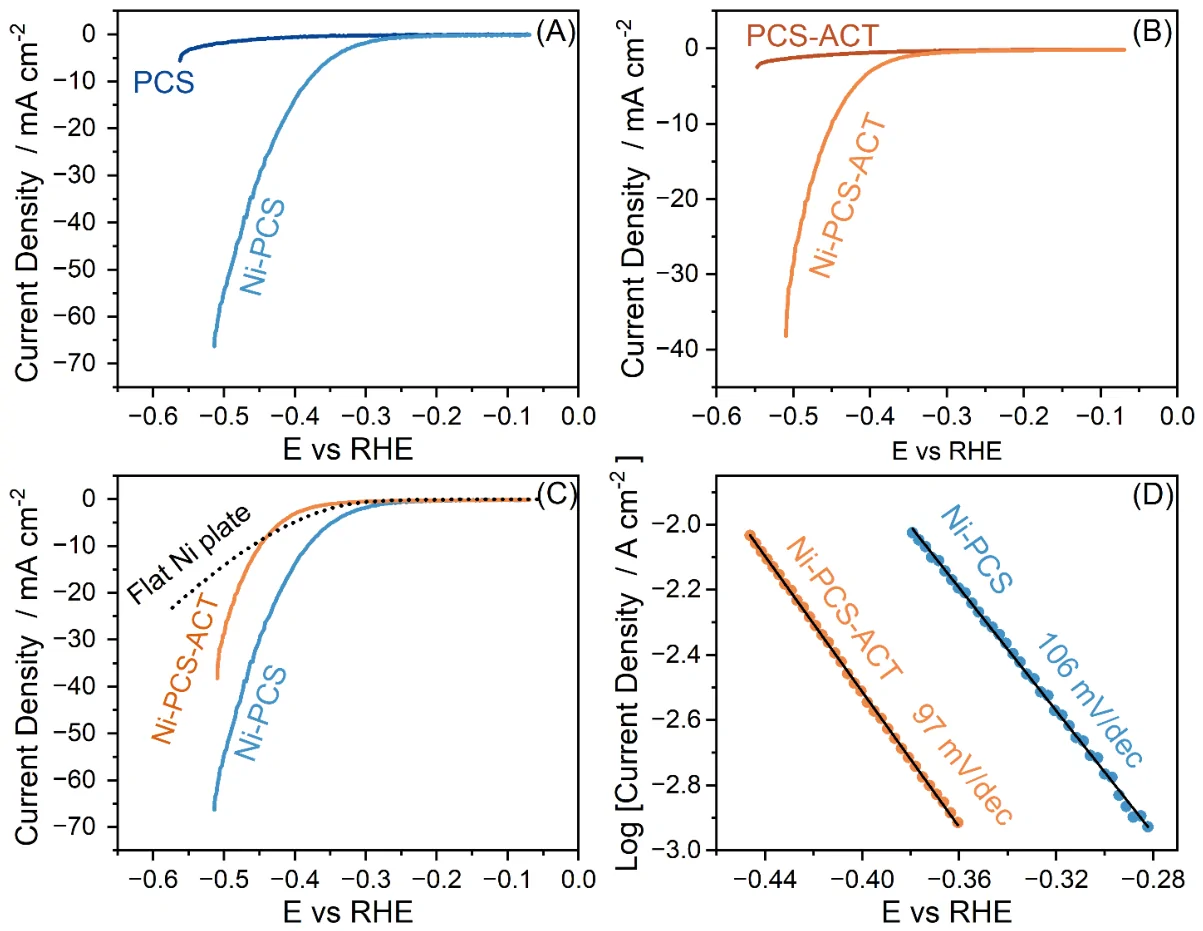
(**A**,**B**) Electrochemical HER performance of PCS, activated PCS (PCS-ACT), nickel on PCS (Ni-PCS), and nickel on activated PCS (Ni-PCS-ACT). (**C**) Comparison of the performance of Ni-PCS and Ni-PCS-ACT with the performance of a flat Ni plate electrode. (**D**) Tafel polarization curves of Ni-PCS and Ni-PCS-ACT. All the curves were recorded in 1M KOH at a scan rate of 1 mVs^−1^.

Linear sweep voltammetry (LSV) was employed to evaluate the electrocatalytic activity of the synthesized materials in the HER by recording the polarization curves (Figure 6). With an increase in cathodic potential, it is expected to see more H_2_ produced, which is manifested as an increase in current density towards more cathodic (negative) values. As seen in Figure 6A, when pure PCS is used, this increase occurs at very negative potentials, albeit with a very minor rise in current density. This is expected to see since carbon materials are poor electrocatalyst for hydrogen evolution by water electrolysis. However, when Ni nanoparticles were immobilized on the PCS, the increase in current density (and thus the H_2_ production rate) with an increase in cathodic potential was significant, as evidence by the difference between the two curves in Figure 6A. This is evidence that the presence of Ni on the PCS surface is crucial for the electrochemical production of H_2_ by water electrolysis. A similar behavior could also be seen for Ni nanoparticles immobilized on the activated PCS (Ni-PCS-ACT) in Figure 6B. However, the comparison of the electrocatalytic activity in the HER between Ni-PCS and Ni-PCS-ACT, presented in Figure 6C, reveals that the former electrocatalyst performed better than the latter one. This is in contradiction with the results presented in Table 2, which show that the specific surface area of nickel on the two carbon sphere catalysts is almost the same; one would thus expect the overlap of the two curves in Figure 6C. However, if we refer to the results in Table 1, we can see that the pore diameter of Ni-PCS is larger than that of Ni-PCS-ACT, allowing easier outgassing of produced hydrogen and thus easier access of water/ions to Ni active sites buried deeper in the PCS pores. This, in turn, results in more Ni sites accessible for the HER.

Compared with the literature, Ni electrodeposited on pencil graphite [33], Ni nanoparticles supported on carbon (Ni/C) [34], and nickel nanoparticle/carbon quantum dot hybrids (Ni/CQD) [35] required overpotentials of approximately −210 mV, −213 mV, and −146 mV, respectively, to reach a current density of −10 mA cm^−2^, all of which are lower than that of Ni-PCS (−370 mV). However, Ni-PCS still outperforms the Ni control electrode, which required an overpotential of −440 mV to achieve the same current density (Figure 6C), demonstrating the beneficial effect of decorating porous carbon spheres with Ni nanoparticles under the present experimental conditions. The higher overpotential of Ni-PCS than that of literature-reported Ni/carbon catalysts may be related to the low surface coverage of PCS by Ni (2.7%) and the limited accessibility of catalytically active Ni sites. It should also be noted that a rigorous comparison would require identical Ni loadings, carbon loadings, electrolyte composition, and testing conditions. Nonetheless, the results presented in Figure 6A–C clearly demonstrate the positive role of Ni nanoparticles anchored on PCS in enhancing the HER activity of porous carbon spheres in alkaline media.

To investigate the rate-limiting step in the HER mechanism, Tafel slopes were calculated from Figure 6D for Ni-PCS (106 mV dec^−1^) and Ni-PCS-ACT (97 mV dec^−1^). The obtained values are in accordance with those for Ni NPs on carbon materials reported in the literature [35,36,37,38,39]. The experimentally determined Tafel slope values are lower than the theoretical value of 117 mV dec^−1^ expected for a Volmer rate-determining step at 22 °C, assuming the charge-transfer coefficient of 0.5. This could be due to the potential-dependent coverage of adsorbed *H_ads_* on Ni and contributions from subsequent Heyrovsky kinetics. This interpretation is consistent with reported alkaline HER kinetics on Ni, where Tafel slopes of approximately 100 mV dec^−1^ have been associated with combined Volmer–Heyrovsky kinetics under conditions of high H coverage [38,39]. The slightly lower experimental values can also be rationalized by an effective charge-transfer coefficient higher than 0.5 [40,41].

**Figure 7:**
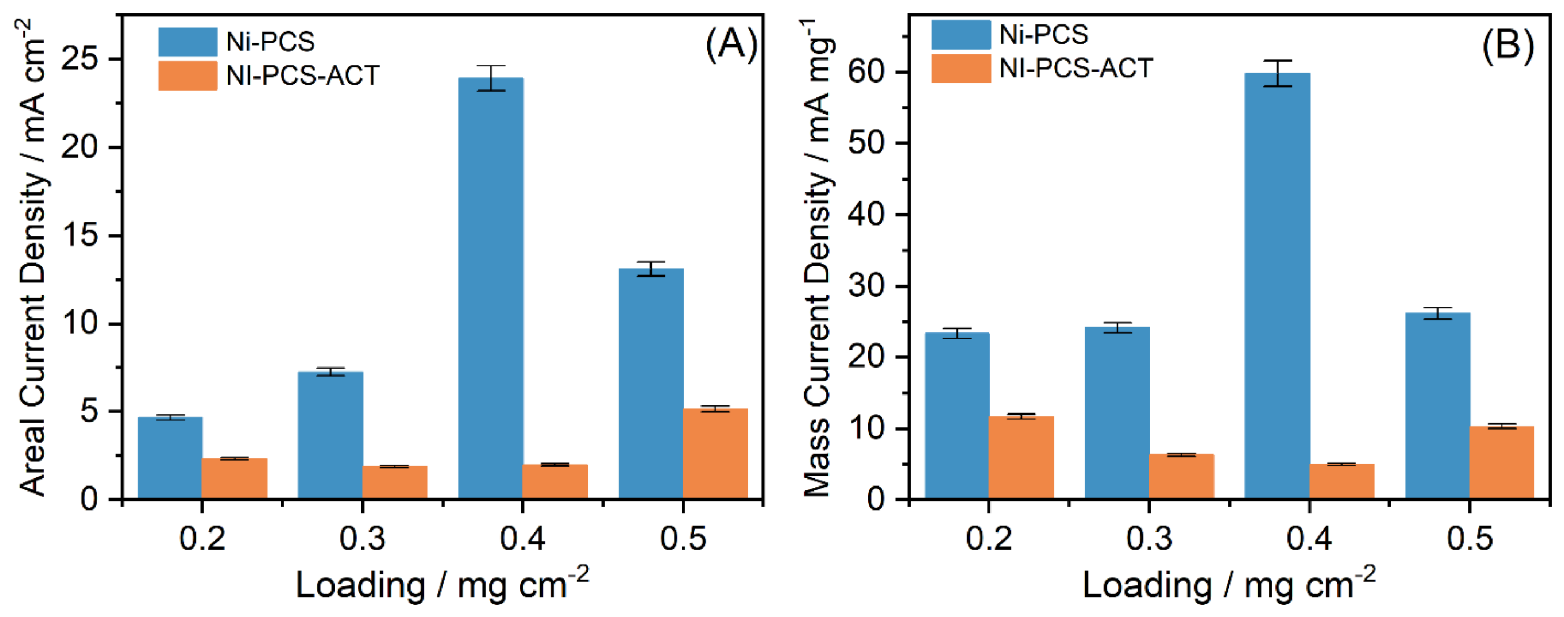
(**A**) Geometric area current density activity of Ni on PCS (Ni-PCS), and Ni on activated PCS (Ni-PCS-ACT) as a function of mass loading of the catalyst obtained from polarization curves at an overpotential of −0.42 V. (**B**) The corresponding mass current density activity of Ni-PCS and Ni-PCS-ACT.

In the experiments presented in Figure 6, the loading of Ni on the cathode was 0.4 mgcm^−2^. However, it is also of interest to investigate how different Ni mass loadings influence the corresponding performance of the cathode in HER, and the results are presented in Figure 7. As shown in Figure 7A, as the mass loading increases, the catalytic activity of Ni-PCS improves up to a peak at 0.4 mg cm^−2^, reaching an areal current density of 24 mAcm^−2^, then declines at 0.5 mg cm^−2^. However, Ni-PCS-ACT exhibits an almost constant areal current density with increasing catalyst loading up to 0.4 mg cm^−2^, and then increases only slightly at a loading of 0.5 mg cm^−2^, still well below the value offered by Ni-PCS at a lower loading (0.4 mg cm^−2^). A similar trend is observed when the current is normalized with respect to ECSA (Figure S4). Thus, when considering both the areal and ECSA-normalized current density (current per geometric or ECSA area of the electrode), the best-performing electrode is Ni-PCS at a loading of 0.4 mg cm^−2^.

Next, the currents were normalized to mass loading to obtain the mass-specific activity in mA mg^−1^ of each sample, as shown in Figure 7B. The loading of 0.4 mg cm^−2^ again yielded the highest catalytic activity for Ni-PCS. However, Ni-PCS-ACT performed the best at loadings of 0.2 and 0.5 mg cm^−2^. Nevertheless, even when the relative comparison of Ni-PCS and Ni-PCS-ACT is made based on mass-specific current density values in Figure 7B, the former electrocatalyst offered better performance at all loadings, which could be due to the better accessibility of Ni sites for the HER and decreased blocking of the catalyst pores by produced H_2_ due to the larger pore diameter of the Ni-PCS electrocatalyst material (see Table 1).

## 3. Materials and Methods

### 3.1. Synthesis of Porous Carbon Spheres (PCSs)

Pluronic F127 [42] (Poly(ethylene glycol)-block-poly(propylene glycol)-block-poly(ethylene glycol) diacrylate, CAS number: 9003-11-6) of 50 mg was dispersed in 60 mL of deionized water. After adding aniline (0.38 mL) and pyrrole (0.29 mL), the solution was stirred for 0.5 h and then left to stand for 1 h at 5 °C (solution A). Next, 1.917 g ammonium persulfate was dissolved in 5 mL deionized water (solution B). Solutions A and B were then cooled to 5 °C and mixed quickly with magnetic stirring. Finally, the mixed solution was placed in an incubator at 5 °C for 24 h. The solid product was thoroughly washed with reverse osmosis (RO) water through vacuum filtration using a mixed cellulose ester (MCE) membrane filter paper (47 mm diameter, 0.65 μm pore size). It was then dried in a vacuum oven at 60 °C to produce the Polymer Hollow Carbon Spheres (PHCSs). Afterwards, 1 g of PHCSs was placed in a tube furnace and heated to 700 °C at a rate of 5 °C min^−1^ under argon gas. Once the temperature reached 700 °C, the sample was maintained at this temperature for 2 h. The final product is referred to as the porous carbon sphere (PCS).

With the aim of increasing hydrophilicity by introducing surface oxygen-containing functional groups, surface defects, and potentially improving Ni nanoparticles anchoring, PCS were also chemically treated (activated). Activated PCS samples were prepared by placing 247 mg of ground PCS in a digestion tube and immersing in a 250 mL beaker filled with ice. The sample was initially treated with deionized water (4.59 mL), followed by the addition of sulfuric acid (13.77 mL) and nitric acid (6.88 mL) (sulfuric acid/nitric acid mixture, a strong oxidizing agent, is used for the chemical modification/functionalization/activation of carbon-based materials [43]), using a micropipette for precise measurements. After adding these reagents, the sample was removed from the ice bath and allowed to equilibrate at room temperature. Subsequently, the sample was digested for 12 h at 50 °C in a hot block. Following digestion, the sample was washed multiple times with deionized water and centrifuged after each wash to remove impurities. The precipitated PCS was then rinsed with reverse osmosis (RO) water until the pH reached 7, and the final volume was adjusted to 15 mL. It was then centrifuged again to precipitate the solid phase. The sample, containing the solid phase and water (separated during the previous centrifugation), was stored in a freezer with dry ice for 24 h. Finally, the sample was freeze-dried for 36 h to obtain activated PCS in powdered form (PCS-ACT).

### 3.2. Immobilization of Ni Nanoparticles on PCS

To form nickel (Ni) nanoparticles on PCS and PCS-ACT, a solution of nickel sulfate hexahydrate (NiSO_4_·6H_2_O) was prepared by dissolving 2.6284 g in 100 mL of RO water to achieve a concentration of 0.1 M [44]. Additionally, a sodium borohydride (NaBH_4_) solution was prepared by dissolving 0.3861 g in 100 mL of RO water to obtain a 0.1 M concentration. 40 mg of ground PCS (or PCS-ACT) was accurately weighed and placed in a 200 mL beaker. A volume of 5 mL of the 0.1 M NiSO_4_·6H_2_O solution was then added to the beaker using a pipette, followed by sonication in an ice bath for 60 min to promote adsorption of nickel ions onto the PCS (or PCS-ACT) surface. Subsequently, the sample was removed from the ice bath for magnetic stirring, and 10 mL of the 0.1 M NaBH_4_ solution was added gradually via pipetting while stirring magnetically. The mixture was then sonicated in the ice bath for an additional 2 h. Finally, the sample was filtered using vacuum filtration with the MCE filter paper and washed with RO water. The sample was at the end dried at 50 °C for 20 to 25 min and labeled (Ni-PCS or Ni-PCS-ACT).

### 3.3. Materials Characterization

Morphological studies of the products were carried out with ultra-high resolution field emission scanning electron microscopy (Hitachi, SU-8000 Cold FE-SEM, Hitachinaka, Japan), using an accelerating voltage of 5.0 kV and an emission current of 10 μA. X-ray diffraction (XRD) was employed to determine the PCS, Ni + PCS and activated samples crystal shape.

The transmission electron microscopy (TEM) analyses were performed using a Thermo Scientific Talos F200X G2 (Brno, Czech Republic), operated at 200 keV. TEM grids were prepared using the drop-cast method, whereby ~10 μL of the samples’ powder dispersed in isopropanol solvent was drop-cast onto a 300 mesh TEM grid and allowed to dry at room temperature. The images obtained in TEM mode were captured using bright-field imaging at different magnifications.

Powder X-ray diffraction (XRD) data was collected on a Bruker D8 Advance diffractometer equipped with a Lynxeye linear position sensitive detector (Bruker AXS, Madison, WI, USA). Samples were smeared directly onto the silicon wafer of a proprietary low-background sample holder. Data were collected using a continuous coupled *θ*/2*θ* scan with Ni-filtered Cu*K*α radiation and operated at 40 kV and 40 mA. Data were collected for an hour between 5 and 90° with an increment of 0.02°.

Raman spectroscopy was used to analyze the degree of graphitization. Raman spectra were obtained using a DXR2 Raman spectrometer (Thermo Fisher Scientific, Waltham, MA, USA) with a 532 nm laser source.

Brunauer–Emmett–Teller (BET) analysis was used to determine the total specific surface area (*S_BET_*) and porosity of both PCS, Ni NPs, and activated PCS-ACT and Ni-PCS-ACT. For PCS, the specific surface area and porous texture were determined using N_2_ adsorption–desorption measurements (Micromeritics TriStar II Plus Version 3.02 analyzer) at −196 °C. Before the adsorption measurements, the samples were degassed at 120 °C for 12 h.

Chemisorption analysis was performed using an Altamira AMI-300 analyzer (Pittsburgh, PA, USA), with H_2_ (99.999%, Linde, Mississauga, ON, Canada) as the probe molecule to estimate the active surface area of Ni NPs via pulse chemisorption. Before pulse chemisorption, we conducted hydrogen temperature-programmed reduction (H_2_-TPR) to investigate the reducibility of NiO nanoparticles and their interaction with the PCS.

To do this, approximately 0.1 g of a sample was placed between layers of quartz wool inside a U-shaped quartz tube. The sample was initially pretreated under a flow of He (99.999% Linde) at a rate of 20 cm^3^ min^−1^, heated to 500 °C at a rate of 5 °C min^−1^, and held at this temperature for 1 h. Subsequently, the sample was cooled to 40 °C before initiating a programmed temperature increase to 800 °C under a flow of 10 vol% H_2_ (99.999%, Linde) in Ar balance. The hydrogen consumed during the reduction process was monitored and quantified using a thermal conductivity detector (TCD). Thereafter, the pulse experiment was conducted using a fresh sample that was pretreated in a He atmosphere at 300 °C for 1 h, then ramped to 550 °C (as determined by H_2_-TPR) under 10% H_2_, and held for 2 h. At this temperature, the flow was switched to Ar, and the sample was flushed for 3 h to clean the sample’s surface. Following this, the sample was cooled to 40 °C under Ar flow, and then a pulse chemisorption was carried out for 20 pulses.

Thermogravimetric analysis (TGA) was conducted using a Netzsch TG 209 F1 Libra instrument (Selb, Germany) to assess the material’s stability. Approximately 15 mg of the sample was placed in a TGA crucible and heated at a rate of 5 °C min^−1^ to 70 °C, where it was held for 1 h under Ar atmosphere.

### 3.4. Electrode Preparation

A mixture of 40 mg of Ni + PCS powder (or only PCS for control experiments) was dispersed in a vial containing 5 mL of 100% ethanol (in electrocatalyst-loading experiments, the weight of Ni + PCS powder was varied from 20 to 50 mg). The dispersion was sonicated in an ice bath for 15 min to ensure uniform dispersion of the solid phase and to deagglomerate nanoparticles. Following this, 14 µL of aquivion binder D79-25BS (25% of the solution) was added into the mixture, and the sample was further sonicated in the ice bath for an additional 0.5 h to promote effective dispersion. Subsequently, 10 µL of the prepared ink was deposited onto a glassy carbon electrode (0.2 cm^2^) and then dried at 30 °C.

### 3.5. Electrochemical Characterization

Linear Polarization (LP) was employed in a traditional three-electrode cell. All three electrodes (working, counter, and reference) were immersed in the same electrolyte (1 M aqueous KOH), while the counter electrode (CE) was separated by a glass frit to prevent oxygen produced at CE to reach a working electrode and interfere with HER [45]. A glassy-carbon working electrode (GCE, surface area: 0.2 cm^2^) with an electrocatalyst layer, a Pt counter electrode and a Hg/HgO reference electrode (filled with 1M KOH) were used. All potentials reported in this study are expressed with respect to the reversible hydrogen electrode (RHE). The electrode potential was corrected for the ohmic drop due to solution resistance using iR compensation. All the electrochemical measurements were performed at a temperature of 22 ± 1 °C.

## 4. Conclusions

Porous carbon sphere (PCS) catalyst support for nickel nanoparticles has been successfully synthesized, followed by its decoration with nickel nanoparticles (Ni NPs) that served as an electrocatalyst for hydrogen production by water electrolysis in an alkaline medium. SEM and TEM analyses revealed that the Ni NPs tended to aggregate into large clusters, resulting in a broad particle size distribution. Moreover, the carbon support exhibited a porous spherical morphology, without the structural confinement typically associated with a porous architecture. Structural characterization by XRD and Raman spectroscopy confirmed that both PCS and the activated PCS (PCS-ACT) possessed predominantly amorphous structures with a significant degree of defects, which are beneficial for anchoring metal nanoparticles. Although the specific surface area (*S_BET_*) of the pristine PCS was found to be significantly higher than that of PCS-ACT, the specific surface area of electrocatalytically active nickel PCS was very close to that of nickel on PCS-ACT. Nevertheless, the electrocatalytic performance of Ni-PCS was superior to that of Ni-PCS-ACT, which was due to the better accessibility of Ni sites for the HER and decreased blocking of the catalyst pores by produced H_2_ due to the larger pore diameter of the Ni-PCS electrocatalyst material. While the Ni–PCS composite demonstrated promising catalytic activity toward HER in an alkaline medium, a performance gap remains when compared with state-of-the-art catalysts reported in the literature.

The principal contribution of this work is the demonstration that porous carbon sphere architecture plays a decisive role in determining the HER performance of Ni-decorated carbon electrocatalysts. Although Ni-PCS and Ni-PCS-ACT exhibited nearly identical electrochemically active Ni surface areas, the non-activated PCS support provided superior HER activity due to improved accessibility of Ni active sites and more effective removal of evolved hydrogen. These results indicate that optimization of pore structure and mass-transport characteristics may be as important as increasing metal dispersion when designing porous carbon-supported Ni electrocatalysts for alkaline water electrolysis.

## Figures and Tables

**Table 1:** Textural properties of PCS, PCS-ACT, Ni-PCS, and Ni-PCS-ACT samples.

Sample	*S_BET_* (m^2^ g^−1^) ^a^	*V_T_* (cm^3^ g^−1^) ^b^	*V_micro_* (cm^3^ g^−1^) ^c^	*V*_meso-macro_ (cm^3^ g^−1^) ^d^	*D_P_* (nm) ^e^
PCS	284 ± 10	0.28	0.12	0.16	4.0
PCS-ACT	143 ± 5	0.32	0.05	0.27	8.9
Ni-PCS	182 ± 6	0.25	0.07	0.18	5.6
Ni-PCS-ACT	117 ± 4	0.15	0.04	0.11	5.3

^a^ *S_BET_* = BET total specific surface area obtained from adsorption data in the 0.05–0.3 *p*/*p*^0^ range; all reported data are within ± 10 m^2^ g^−1^ based on repeated analysis. ^b^ Total pore volume (*V_T_*). ^c^ Micropore volume obtained using the *t*-plot method (*V_micro_*). ^d^ Mesopore and macropore volumes (*V*_meso-macro_). ^e^ Calculated via the Barrett–Joyner–Halenda (BJH) method from the adsorption branch of a N_2_ isotherm.

**Table 2:** Results of the H_2_ chemisorption analysis of Ni-PCS and Ni-PCS-ACT.

Sample	Uptake (µmol g^−1^) ^a^	Active Surface (m^2^ g^−1^)
Ni-PCS	126.8 ± 8.4	5.0 ± 0.3
Ni-PCS-ACT	130.0 ± 5.4	5.1 ± 0.2

^a^ The monolayer uptake was calculated from the H_2_ pulse experiment. The uncertainty is based on repeated analysis.

## Data Availability

Data are contained within the article and Supplementary Materials.

## Supplementary Materials

The following supporting information can be downloaded at: https://www.mdpi.com/article/10.3390/molecules31183308/s1. Figure S1: Particle size distribution of Ni nanoparticles immobilized on (A) PCS and (B) PCS-ACT; Figure S2: SAED of (A) Ni-PCS and (B) Ni-PCS-ACT; Figure S3: H_2_ pulse chemisorption of Ni-PCS and Ni-PCS-ACT samples; Figure S4: ECSA-normalized current density activity of Ni on PCS (Ni-PCS) as a function of mass loading of the catalyst obtained from polarization curves at overpotential of −0.42 V.
