# Supplementary material for: Porous Carbon Spheres (PCSs) as a Support for Ni Nanoparticles for Alkaline Water Electrolyzer Cathodes
Source: Molecules. 2026 Sep 18;31(18):3308. doi: 10.3390/molecules31183308 (PMC13610042; doi:10.3390/molecules31183308)
Supplement: Supplementary file 1 [file molecules-31-03308-s001.zip › molecules-4509758-supplementary.pdf]

Supplementary Materials

Porous Carbon Spheres (PCS) as a Support for Ni Nanoparticles for Alkaline Water Electrolyser Cathodes

Ahmed A.A. Almaazmi*, Galal Nasser, Lantao Liu, Jan Kopyscinski, Sasha Omanovic*

Department of Chemical Engineering, McGill University, 3610 University Street, Montreal, Quebec, H3A 0C5, Canada

* To whom all correspondence should be addressed: ahmed.almaazmi@mail.mcgill.ca; sasha.omanovic@mcgill.ca

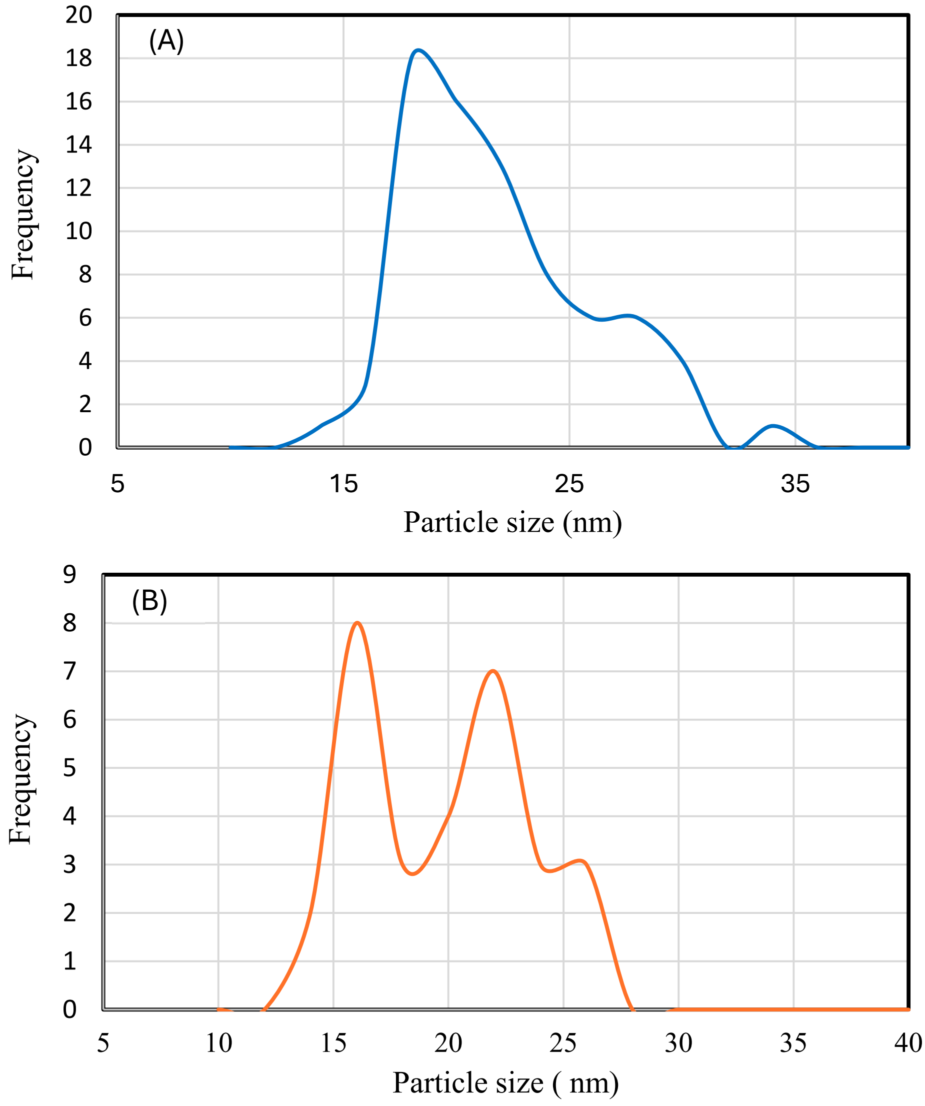

Figure S1: Particle size distribution of Ni nanoparticles immobilized on (A) PCS and (B) PCS-ACT.

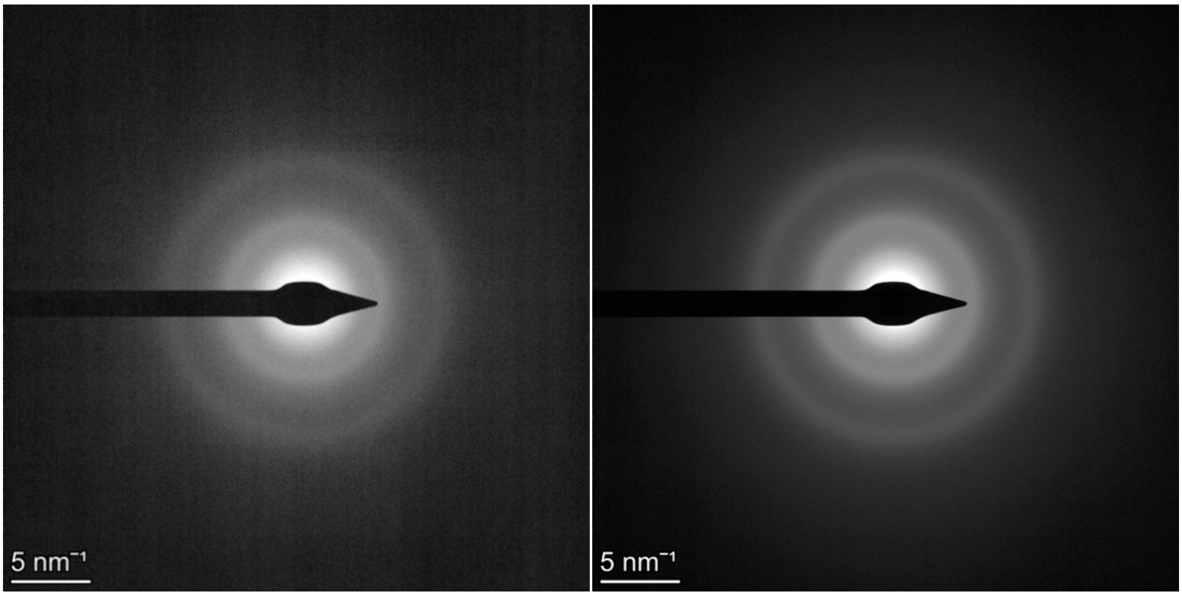

(A) **(B)**

Figure S2: SAED of (A) Ni-PCS and (B) Ni-PCS-ACT

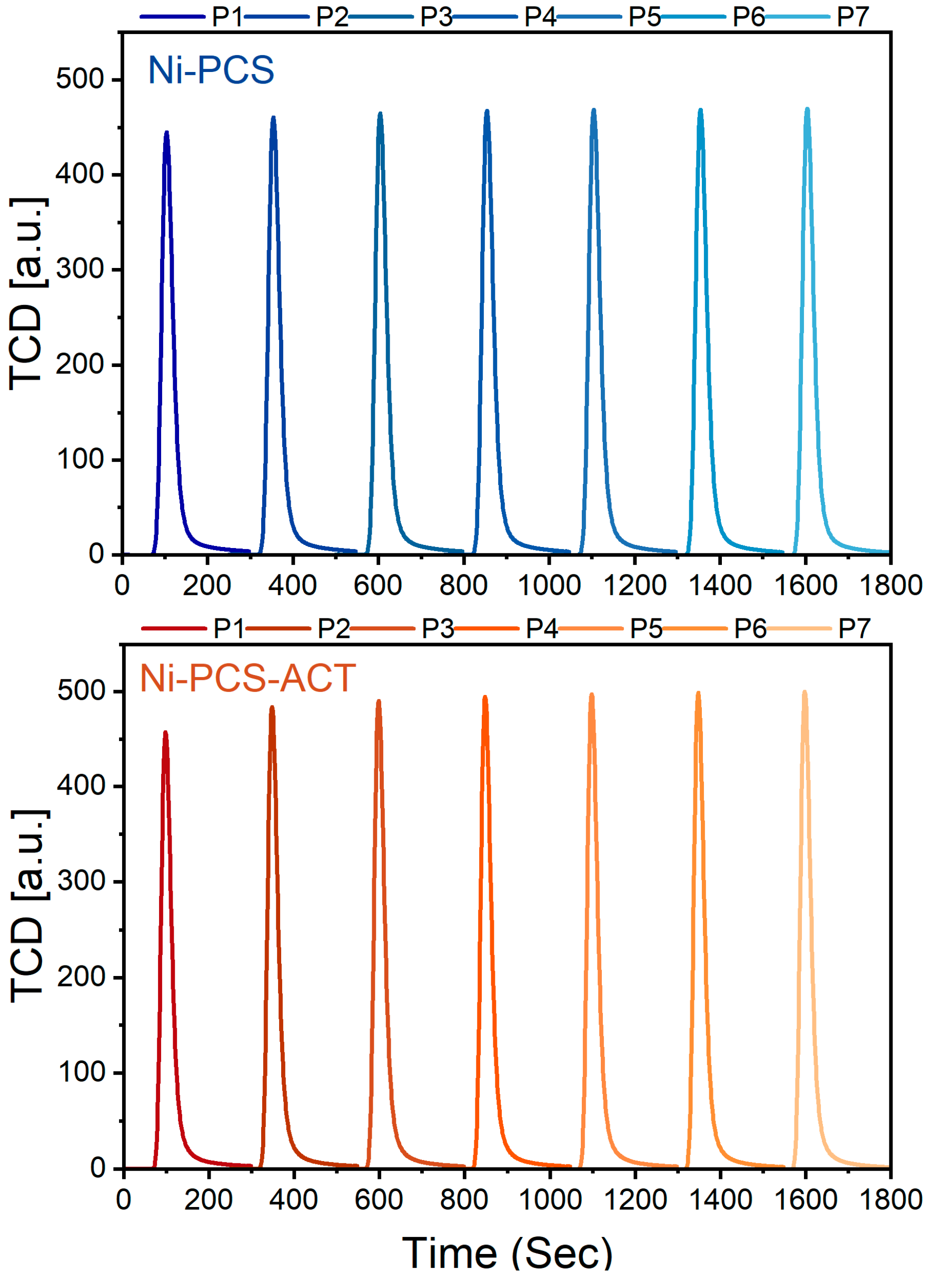

Figure S3: H_2 pulse chemisorption of Ni-PCS and Ni-PCS-ACT samples.

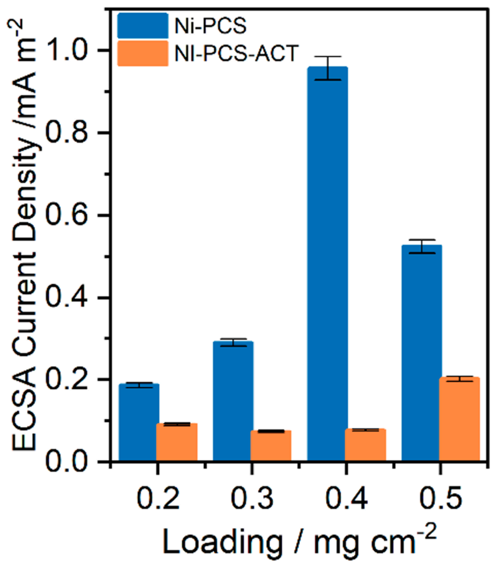

Figure S4: ECSA-normalized current density activity of Ni on PCS (Ni-PCS) as a function of mass loading of the catalyst obtained from polarization curves at overpotential of −0.42 V.
